# Reduced representation sequencing detects only subtle regional structure in a heavily exploited and rapidly recolonizing marine mammal species

**DOI:** 10.1002/ece3.4411

**Published:** 2018-08-05

**Authors:** Nicolas Dussex, Helen R. Taylor, Willam R. Stovall, Kim Rutherford, Ken G. Dodds, Shannon M. Clarke, Neil J. Gemmell

**Affiliations:** ^1^ Department of Anatomy University of Otago Dunedin New Zealand; ^2^ Department of Bioinformatics and Genetics Swedish Museum of Natural History Stockholm Sweden; ^3^ Invermay Agricultural Centre AgResearch Puddle Alley Mosgiel New Zealand

**Keywords:** commercial fisheries, conservation, genotyping‐by‐sequencing, pinnipeds, population genetics, single nucleotide polymorphisms

## Abstract

Next‐generation reduced representation sequencing (RRS) approaches show great potential for resolving the structure of wild populations. However, the population structure of species that have shown rapid demographic recovery following severe population bottlenecks may still prove difficult to resolve due to high gene flow between subpopulations. Here, we tested the effectiveness of the RRS method Genotyping‐By‐Sequencing (GBS) for describing the population structure of the New Zealand fur seal (NZFS,* Arctocephalus forsteri*), a species that was heavily exploited by the 19th century commercial sealing industry and has since rapidly recolonized most of its former range from a few isolated colonies. Using 26,026 neutral single nucleotide polymorphisms (SNPs), we assessed genetic variation within and between NZFS colonies. We identified low levels of population differentiation across the species range (<1% of variation explained by regional differences) suggesting a state of near panmixia. Nonetheless, we observed subtle population substructure between West Coast and Southern East Coast colonies and a weak, but significant (*p* = 0.01), isolation‐by‐distance pattern among the eight colonies studied. Furthermore, our demographic reconstructions supported severe bottlenecks with potential 10‐fold and 250‐fold declines in response to Polynesian and European hunting, respectively. Finally, we were able to assign individuals treated as unknowns to their regions of origin with high confidence (96%) using our SNP data. Our results indicate that while it may be difficult to detect population structure in species that have experienced rapid recovery, next‐generation markers and methods are powerful tools for resolving fine‐scale structure and informing conservation and management efforts.

## 1 INTRODUCTION

Population genetic analyses have become an integral part of conservation studies. These analyses allow the quantification of parameters relevant to endangered populations such as genetic diversity, effective population sizes, gene flow and the inference of past population histories (Beaumont, Zhang, & Balding 2002; Lopes & Boessenkool, 2010). Identifying management units and determining the degree of connectivity among demes are often the first steps in allocating conservation resources and developing management strategies (Moritz, 1999; Palsbøll, Bérubé, & Allendorf, 2007; Schwartz, Luikart, & Waples, 2007). For example, species with a relative lack of population structure that show little variation across their ranges benefit from management of all subpopulations as a single unit (Palsbøll et al., 2007). Conversely, species characterized by genetically‐distinct, geographically isolated demes require management of distinct units to maximize maintenance of the species’ evolutionary potential (Lowe & Allendorf, 2010; Palsbøll et al., 2007).

While describing population structure is crucial for species management, considering the underlying causes of this structure and discriminating between events of evolutionary significance and those with anthropogenic causes is also critical (e.g., rock wren; Weston & Robertson, 2015). For instance, if genetically distinct populations have diverged gradually as the result of glacial isolation or post‐glacial colonization (e.g., kea; Dussex, Wegmann, & Robertson, 2014), it is often considered best to work toward maintaining that existing population structure. Conversely, for species that have undergone severe reductions in population size and genetic population fragmentation (e.g., mohua; Tracy, Wallis, Efford, & Jamieson, 2011), restoration of populations to better reflect the population structure prior to human disturbance may be more beneficial (Moritz, 1994; Palsbøll et al., 2007).

Recent advances in next‐generation sequencing (NGS) allow the characterization of large numbers of molecular markers, increasing the resolution of comparisons of individuals within and between populations (Helyar et al., 2011). Single nucleotide polymorphisms (SNPs) have become the markers of choice for an increasing number of modern population genetic studies (Andrews, Good, Miller, Luikart, & Hohenlohe, 2016; Helyar et al., 2011), despite initial speculation on their relative lack of genetic diversity compared to individual microsatellites (Rosenberg, Li, Ward, & Pritchard, 2003). While 100 SNPs may only be as informative as 10–20 microsatellites (Kalinowski, 2002), tens of thousands of SNPs can be discovered via NGS, even for non‐model species (Ellegren, 2014; Seeb et al., 2011). The degree of definition afforded by such large marker datasets therefore statistically outperforms studies in which a few dozen microsatellites were used to describe population structure (Helyar et al., 2011; Liu, Chen, Wang, Oh, & Zhao, 2005).

A growing number of studies have also explored the specific applications of reduced representation sequencing (RRS) methods in evolutionary biology (e.g., RADseq; Andrews et al., 2016). One of these methods, referred to as Genotyping‐by‐sequencing (GBS; Elshire et al., 2011) has been used in a range of non‐model species as a means of inferring genotypes and describing population genomic structure (Johnson et al., 2015; Skovrind et al., 2016). Species for which traditional markers (i.e., microsatellite, single mtDNA sequences) have shown limited resolution in the description of population structure (e.g. Dussex et al., 2016; Robertson & Gemmell, 2005) could benefit from RRS methods, due to the higher definition and overall performance of the resulting marker sets. For instance, species characterized by strong dispersal potential and high gene flow are interesting candidate species for re‐evaluations of population structure analyses using RRS.

The New Zealand fur seal (NZFS, *Arctocephalus forsteri*) has undergone a rapid recolonization following near extinction. Prior to the arrival of humans, NZFS were distributed throughout the east and west coasts of New Zealand's North and South Islands (Smith, 1989, 2005). Pre‐historic Polynesian settlers hunted NZFS as a source of meat, which resulted in a rapid population decline (Emami‐Khoyi et al., 2017; Smith, 1989, 2005) and the near elimination of the mainland fur seal population (Lalas & Bradshaw, 2001). By the time of European arrival in New Zealand in the late 18th Century, the NZFS mainland breeding range was confined to the south‐western South Island (Smith, 1989). Sealing by European settlers for the acquisition of furs had a further devastating impact on NZFS and resulted in a depletion of the NZFS population (Lalas & Bradshaw, 2001). Overall, an estimated 1.5 million skins were harvested between 1792 and 1849 around Australia's southern coast, New Zealand, and at the adjacent subantarctic islands (Ling, 1999), suggesting that the number of NZFS before sealing may have been as high as 1–2 million (Baird, 2011). However, because records of the number of fur seals killed during that time are incomplete, inaccurate, or deliberately misleading to protect commercial interest of the day, it is unclear how many seals may have remained in New Zealand in the 1830s, when sealing stopped due to low profitability (Lalas & Bradshaw, 2001).

**Figure 1:**
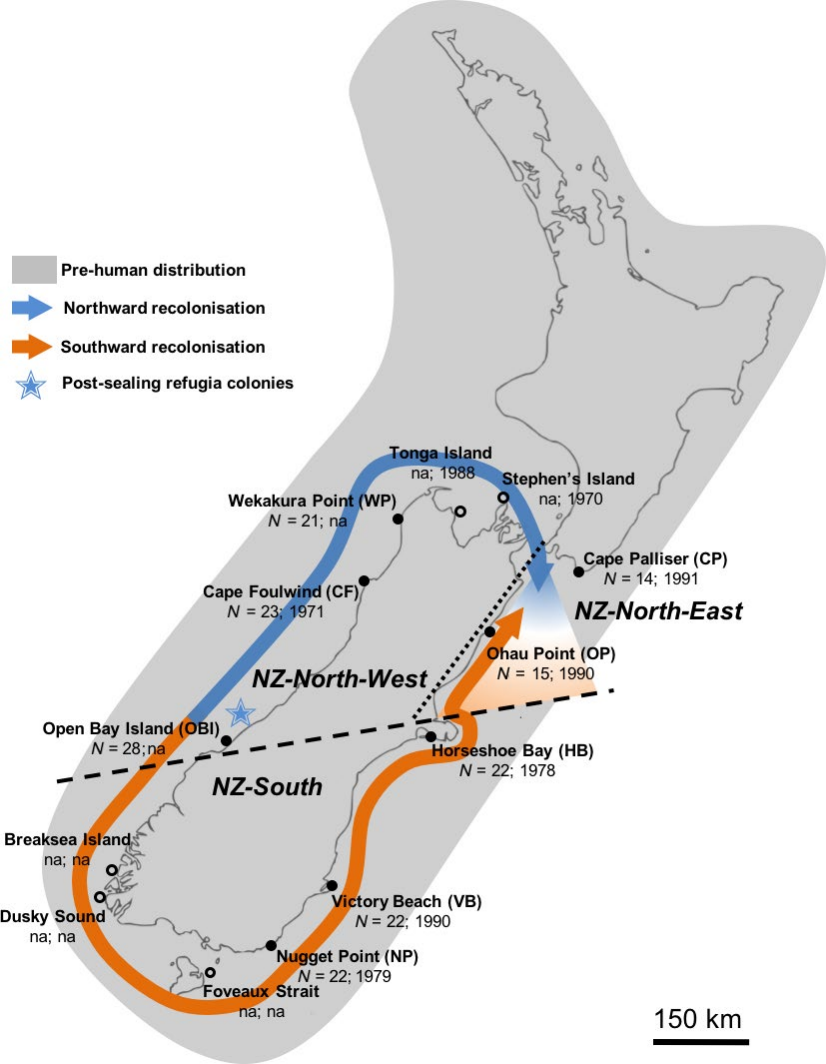
Extant New Zealand fur seal (*A. forsteri*) breeding colonies with full and empty dots depicting colonies sampled and not sampled in this study, respectively, with the number of samples analysed in this study and the year of first breeding from King (2005). Cape Palliser (CP) is currently the only known breeding colony in the North Island. The dashed line represents the genetic clusters based on Dussex et al. (2016) and Salis et al. (2016) microsatellite and mtDNA results, and the dotted line represents the approximate limit of the admixture zone between the two main clusters. Arrows depict the post‐sealing recolonization scenario from hypothetical south‐western South Island refugia depicted with a star inferred by ABC analyses (Dussex et al., 2016; this study) [Colour figure can be viewed at http://wileyonlinelibrary.com]

Since implementation of regulations in the late 19th century and the official cessation of commercial sealing in 1946 (Sorensen, 1969), the NZFS has rapidly recolonized much of its former range, and has re‐established breeding colonies in the South Island of New Zealand (Baird, 2011; Bradshaw, Lalas, & Thompson, 2000; Crawley & Wilson, 1976). However, only one breeding colony, founded in the early 1990s, has been re‐established in the North Island (Dix, 1993). Many of these colonies are showing steady population growth (i.e., 20%–25% annual growth) (Lalas & Bradshaw, 2001; Boren, Muller, & Gemmell, 2006; Figure 1). The current NZFS population is estimated at 50,000–100,000 animals although there is great uncertainty around this number (Baird, 2011).

Population declines and fragmentation often lead to population differentiation via the effect of genetic drift when demographic units within a larger panmictic population become fixed for different alleles (Charlesworth, 2009; Frankham, 2005). Alternatively, genetic differentiation can arise via founder effects following the recolonization of a species’ former range (Ramachandran et al., 2005; Templeton, 1980). However, previous studies on pinniped species with histories of intense exploitation have shown a lack of (e.g., *Arctocephalus gazella*, Hoffman, Grant, Forcada, & Phillips, 2011) or only moderate fine‐scale structure (e.g., *Arctocephalus tropicalis*, Wynen et al., 2000; *Halichoerus grypus*, Fietz et al., 2016). Research based on modern and ancient DNA suggests that, while there was a shift in the genetic composition of NZFS in southern New Zealand resulting from the demographic decline, this decline may not have been long enough to induce a significant loss of genetic diversity as evidenced by comparable levels of genetic diversity in modern and ancient populations (Salis et al., 2016). This result is in stark contrast with the severe demographic decline and near range‐wide extirpation in the 1800s. Moreover, in spite of potential founder effects resulting from recolonization from a few “refugia” colonies, studies based on microsatellite data have suggested that any population differentiation would have been transient, owing to the high vagility of the species combined with a rapid recolonization time (Figure 1, Dussex et al., 2016).

Studies aimed at assigning NZFS individuals to their colonies or broader regions of origin using microsatellite markers and classical assignment approaches have also had limited success (Dussex et al., 2016; Robertson & Gemmell, 2005). Genetic distinction of potential management units has thus been difficult. A prevailing view from the literature published on NZFS genetics to date is that more numerous and/or variable genetic markers could significantly aid in resolving structure and improving assignment probabilities (Dussex et al., 2016; Robertson & Gemmell, 2005)—using an RRS approach provides the opportunity to empirically test this prediction.

Today, NZFS are the most commonly caught marine mammal in New Zealand commercial trawl net fisheries (Thompson & Abraham, 2010). Between 2003 and 2013 there were an estimated 501–1,273 fur seals caught per fishing season (Ministry for Primary Industries 2014; Thompson & Abraham, 2010) and males accounted for about 45% of reported bycaught individuals (Baird, 2011). Bycatch incidents seem to occur in clusters throughout New Zealand waters, mostly off the northern East (~40%) and West Coasts of the South Island and around offshore islands (Thompson & Abraham, 2010). While it is possible that bycatch may affect some colonies more than others, there is a high degree of uncertainty on the localized impact of commercial fisheries on the species. West Coast colonies in particular face the challenges of lower habitat accessibility (Smith, 1989, 2005), climatic shifts that affect food availability (Bradshaw, Lalas, et al., 2000) and potentially higher conflict with fisheries (Ministry for Primary Industries, 2014). Moreover, the possibility of sexual segregation and niche divergence as seen in the country's other native pinniped, the New Zealand sea lion (*Phocarctos hookeri*) (Leung, Chilvers, Nakagawa, Moore, & Robertson, 2012), could result in sex‐biased mortality which would then negatively impact population dynamics. This means that in spite of an overall trend in steady growth, these effects may negatively affect colony viability. The ability to assign bycaught individuals to their colonies of origin could thus help identify the colonies most at risk of decline.

In this study, we use SNP markers discovered via GBS to examine the population structure in NZFS and compare the markers’ performance with the traditional markers used in previous studies on the same species (Dussex et al., 2016; Lento, Haddon, Chambers, & Baker, 1997; Robertson & Gemmell, 2005). We also assess the power of GBS‐generated SNPs to assign potential bycatch to their colony by estimating the proportion of pups that can be correctly assigned to their known colony and/or regions of origin using a GBS SNP dataset. We hypothesize that in spite of the high individual vagility and rapid recolonization potential of the species, GBS‐generated SNPs will allow the detection of fine‐scale population differentiation among colonies. Moreover, we hypothesize that the SNPs presented here will outperform previously used microsatellite markers and produce more confident assignment probabilities.

## 2 MATERIALS AND METHODS

### 2.1 Sampling

Between 1998 and 2003, we obtained 169 NZFS skin samples from eight mainland NZFS breeding colonies (Figure 1). Only pups were sampled because they are a direct representation of each breeding colony, and to avoid bias caused by seasonal and yearly variation in adult dispersal. For each pup sampled, a small piece of skin was taken from the tip of a digit on a hind flipper using piglet ear notch pliers as per an established protocol (Majluf & Goebel, 1992) and stored in 70% ethanol.

### 2.2 DNA extraction, library preparation, and sequencing

DNA was extracted from fur seal skin samples using a modified version of a lithium chloride DNA extraction protocol (Gemmell & Akayima, 1996). DNA quality and concentration were evaluated using both a Nanodrop 2000c spectrophotometer (Thermo Fischer Scientific) and a Fluorometric Quantitation Qubit system (Thermo Fischer Scientific). For library preparation, we used GBS as a sequencing preparation protocol (Elshire et al., 2011). We used two 96‐well plates for library construction. Prior to adapter ligation, an automated picogreen assay (Thermo Fisher Scientific) was performed to normalize the DNA concentration in all samples to 20 ng/μl. The restriction enzyme *Pst1* was used due to its empirical success for sequencing of vertebrates and its capacity to produce libraries with high depth of coverage (De Donato, Peters, Mitchell, Hussain, & Imumorin, 2013). 17 μl H20, 2 μl CutSmart buffer, 0.5 μl NEB buffer, 0.5 μl *Pst*1 were added to wells containing DNA and adapters. A solution containing ligase buffer, ATP, and T4 ligase was then added to each well to aid in adhering adapters to sticky ends of the now‐cut fragments. This combination of DNA, enzyme, adapters, and ligation solution was incubated at 22°C for 1 hr, and the temperature was then increased to 65°C for 30 min to inactivate T4 ligase (Elshire et al., 2011). 5 μl from each well were pooled together into one solution containing all fragments, each of which was identifiable by its respective barcode adapter. Fragments were amplified in three subsequent polymerase chain reactions and fragment length (in base pairs) was examined on a bioanalyser (Agilent 2100 bioanalyser, Santa Clara, CA, USA). The amplified library was purified using a Pippin Prep (SAGE Science, Beverly, MA, USA) to select the DNA sequencing library in the size range of 150–500 bp. Finally, single‐end sequencing (1 × 100) was performed on an Illumina HiSeq2500 using v4 chemistry, which yielded approximately 25 Gb of raw sequence data per lane.

### 2.3 SNP calling and filtering

SNP calling was performed in Stacks v. 1.30 (Catchen et al., 2011). We used “*process_radtags*” to demultiplex the data and discard low quality data with a probability of sequencing error >0.10% (Phred score = 10). SNP loci were then called using the Stacks “*denovo_map.pl*” pipeline (i.e., without a reference genome), using a minimum stack depth (‐m) of 5 and default parameters for mismatches between loci within samples (‐M = 2) and when building the catalog (–*n* = 1). The “populations” program in Stacks was used to filter out monomorphic loci and sort individuals into separate files for each colony. 180,303 biallelic SNPs remained after this filtering. PLINK 1.9 (Purcell et al., 2007) was then used to exclude individuals with >90% missing data (–mind filter), SNPs with >20% missing call rates (–geno filter), and SNPs with minor allele frequency <5% (–maf filter). SNPs significantly deviating (exact test) from Hardy–Weinberg equilibrium (HWE) (–hardy filter) and pairs of SNPs in linkage disequilibrium (LD) (–ld filter) in at least six of our eight populations were removed. For downstream analyses, we used the whole set of SNPs generated in PLINK, but also generated a separate subset of 5,000 and 1,000 random SNPs for computationally intensive analyses (e.g., STRUCTURE, DIYABC). Finally, the resulting. Ped files were converted into other file formats with the program PGDSPIDER 2.0 (Lischer & Excoffier, 2012), enabling their input into relevant population genetic analysis software.

### 2.4 Genetic diversity and population structure

For our analyses of population structure, we evaluated genetic variation at the colony and genetic cluster levels. Three main clusters were defined based on previous NZFS genetic studies (Dussex et al., 2016; Salis et al., 2016) as well as the results presented here: NZ‐North‐West (OBI: Open Bay Island; CF: Cape Foulwind; WP: Wekakura Point), NZ‐North‐East (CP: Cape Palliser; OP: Ohau Point), previously identified as a zone of admixture (Dussex et al., 2016) and NZ‐South (East Coast to south coast colonies; HB: Horseshoe Bay; VB: Victory Beach; NP: Nugget Point) (Figure 1).

Genetic diversity (*H*_o_, *H*_e_ and *F*_IS_) was estimated for each colony and region using Arlequin v. 3.5 (Excoffier & Lischer, 2010). Pairwise *F*_ST_ was calculated among the eight colonies in Arlequin v. 3.5 using Slatkin's linearized *F*_ST_ (Slatkin, 1995) and tested for Isolation by distance (IBD) using a Mantel test implemented in GenAlEx (Peakall & Smouse, 2006). We then tested for correlations between estimates of *H*_e_ and *F*_ST_ derived from microsatellite loci by Dussex et al. (2016) and the biallelic SNPs genotyped here. For *H*_e_, we calculated the Pearson correlation coefficient (“cor.test” in R; R Development Core Team 2012) and for *F*_ST_, we used a Mantel test for matrix correspondence for correlation (“mantel.rtest” in R; R Development Core Team 2012).

We used the Bayesian cluster methodology implemented in STRUCTURE 2.2 (Falush, Stephens, & Pritchard, 2003; Pritchard, Stephens, & Donnelly, 2000) to make inferences regarding the number of population clusters in our sample of NZFS using a random subset of 5,000 SNPs generated with PLINK 1.9 (Purcell et al., 2007). We used an admixture model with correlated allele frequencies and performed 10 iterations (chain length 500,000 steps, burn‐in = 200,000 steps) for each *K* (from 1 to 8), using the LOCPRIOR option to assist with clustering of faint‐structure data (Hubisz, Falush, Stephens, & Pritchard, 2009). The number of distinct genetic clusters were inferred using the LnP(*K*) value of STRUCTURE 2.2 and the ∆*K* (Evanno, Regnaut, & Goudet, 2005) method implemented in STRUCTURE HARVESTER (Earl & vonHoldt, 2012). CLUMPAK (Kopelman, Mayzel, Jakobsson, Rosenberg, & Mayrose, 2015) was then used to estimate individuals’ assignment coefficient (q) to each genetic cluster and to visualize the results. As a comparison, we also used the program ADMIXTURE (Alexander, Novembre, & Lange, 2009) to estimate the number of clusters for each *K* (from 1–8). This program estimates ancestry in a model‐based manner where individuals are considered unrelated and uses a cross‐validation procedure to determine the best number of possible genetic groups present in the dataset.

STRUCTURE and ADMIXTURE results can be biased in nonequilibrium populations and result in an upward bias in the estimation of *K* because the program relies on assumptions of HWE and linkage equilibrium within populations for each *K* tested (Kaeuffer, Réale, Coltman, & Pontier, 2007). The population structure was therefore also examined independently using a discriminant analysis of principal components (DAPC; Jombart, Devillard, & Balloux, 2010), in *adegenet* (Jombart & Ahmed, 2011) for R (R Development Core Team 2012). DAPC has been shown to perform as well as STRUCTURE and has the advantage of being unconstrained by the assumptions of HWE (Jombart et al., 2010). DAPC is similar to principal component analysis, but unlike PCA, which maximizes the total variation in the dataset, DAPC maximizes the variation among different groups and minimizes variation within groups (Jombart et al., 2010). This analysis was performed with prior information on individual colonies. Finally, analyses of molecular variance (AMOVA; Excoffier, Smouse, & Quattro, 1992) were performed in Arlequin v. 3.5 for (a) a two‐cluster grouping: NZ‐North (OBI, NP, WP, CP, OP) vs. NZ‐South (HB, VB, NP), and (b) a three‐cluster grouping: NZ‐North‐West (OBI, NP, WP) vs. NZ‐North‐East (CP, OP) vs. NZ‐South (HB, VB, NP).

### 2.5 Demographic history

Previous studies have established several aspects of NZFS population history using Approximate Bayesian Computation (ABC; Beaumont, Zhang, & Balding 2002) approaches with microsatellite (Dussex et al., 2016) and mitogenome data (Emami‐Khoyi et al., 2017). Here, we did not attempt to reiterate the demographic model comparisons described in these studies, but rather to estimate demographic parameters based on SNP data for the most likely scenarios inferred in these studies. We thus designed a scenarios including a Polynesian‐induced decline (~500–1,000 years ago; Emami‐Khoyi et al., 2017), and a European‐induced decline (~125–250 years ago; Dussex et al., 2016) followed by range‐wide recolonization from a single refugium and subsequent population divergence with a NZ‐North (OBI, CF, WK, CP, OP) and NZ‐South cluster (HB,VB,NP) (Dussex et al., 2016; Salis et al., 2016) (Supporting Information Figure S1, Table 1). We also designed a null model of constant effective population size though time followed by the same population divergence. The generation time for NZFS is estimated at 4–6 years in females and at 5–9 years in males (Dickie & Dawson, 2003). Thus, we assumed an average 7 year generation time for NZFS.

**Table 1:** Prior and posterior distributions of parameters for a demographic scenario including a Polynesian‐ and European‐induced decline followed by recolonization and population divergence that obtained the highest posterior probability (PP = 1) when comparing two demographic scenarios in New Zealand fur seal (*A. forsteri*). Timing of events is in generations, assuming a generation time of 7 years (Dickie & Dawson, 2003). Type I and II errors were of 0.04 and 0.05, respectively

Parameter	Prior distribution	Posterior mode	5%	95%
*N*_*e‐NZ‐S*_	Uniform [10^2^–10^4^]	1,120	483	7,940
*N*_*e‐NZ‐N*_	Uniform [10^2^–10^4^]	652	336	6,600
*N*_*e‐refugium*_	Uniform [10^2^–2.5 × 10^3^]	110	102	161
*N*_*e‐historical‐NZ*_	Uniform [10^3^–5 × 10^6^]	572,000	228,000	4,750,000
*N*_*e‐pre‐historical*_	Uniform [10^3^–5 × 10^6^]	4,900,000	356,000	4,810,000
*t‐Polynesian*	Uniform [100–200]	106	105	194
*t‐Europeans*	Uniform [25–50]	49.2	30.2	49
*t‐post‐seal*	Uniform [1–25]	20.3	5.89	24.2
*Conditions: t‐Europeans>t‐postseal; N*_*e‐pre‐historical*_ *>N*_*e‐NZ‐N*_ *; N*_*e‐pre‐historical*_ *>N*_*e‐NZ‐S*_				

Because DIYABC is a computationally intensive method, analyses were performed using a subset of 1,000 randomly selected SNPs from our complete dataset. We generated 10^6^ simulations for each scenario. As summary statistics, for single sample statistics we used the mean gene diversity across polymorphic loci and mean gene diversity across all loci (Nei, 1987). For two sample statistics, we used mean of nonnull *F*_ST_ distances between the two samples across loci, the mean of *F*_ST_ distances between the two samples across all loci, mean of nonnull Nei's distances between the two samples across loci, and mean of Nei's distances between the two samples across all loci (Nei, 1972). We used the standard Hudson's (2002) algorithm for selection of minimum allele frequency (MAF).

The posterior probability of each scenario was estimated using both the direct and logistic regression approaches (Fagundes et al., 2007). The ten thousand datasets (1%) with the smallest Euclidean distances were then retained to build posterior parameter distribution. To check model performance, we first empirically evaluated the power of the model to discriminate among scenarios (confidence in scenario choice). The approach consists of simulating pseudo‐observed datasets with parameters drawn from the posterior parameter distribution of the considered scenario and positioning the summary statistics of the observed data in the summary statistic distribution of these pseudo‐observed data. The scenario is then considered suitable if the observed data summary statistics are included in the confidence interval drawn from pseudo‐observed data. We calculated statistical measures of performance and Type I and Type II error rates as a means of model checking (Cornuet, Ravigné, & Estoup, 2010; Excoffier, Estoup, & Cornuet, 2005).

### 2.6 Outlier loci detection

To maximize assignment power to the colonies sampled, we attempted to identify colony‐specific and genetic clusters‐specific sets of outlier loci that showed high differentiation between colonies and genetic clusters. These analyses were performed for each of the eight colonies and for the three genetic clusters.

We identified outlier loci using two different methods. First, we ran Fdist2 (Beaumont & Nichols, 1996) with 50,000 simulations, a confidence interval of 0.99, and false discovery rate (FDR) of 0.05 in LOSITAN (Antao, Lopes, Lopes, Beja‐Pereira, & Luikart, 2008). To correct for multiple testing, we transformed the *p*‐values obtained to *q*‐values (i.e., the false discovery rate FDR, analog to the *p*‐value; Benjamini & Hochberg, 1995). Secondly, we used a Bayesian simulation‐based test (Beaumont & Balding, 2004) that has been further refined and implemented in the software Bayescan 2.0 (Foll & Gaggiotti, 2008). We based our analyses on 10‐pilot runs each consisting of 5,000 iterations, followed by 100,000 iterations with a burn‐in of 50,000 iterations. We used a FDR of 0.05 as the threshold for outlier locus detection in this test.

### 2.7 Population assignments

To assess the power of GBS‐generated SNPs to assign potential NZFS bycatch to their colony or genetic cluster, we treated pups as unknown in origin and attempted to assign them back to their home colonies and genetic clusters. We first performed assignments of pups to the eight colonies using all 26,026 SNPs, a random subset of 5,000 SNPs and the 74 colony‐specific outlier loci. Secondly, we performed assignments of pups to the three genetic clusters using all 26,026 SNPs, the same random subset of 5,000 SNPs and 18 genetic cluster‐specific outlier loci (see Results). This made for a total of six analyses.

We used Genodive 2.0b23 (Meirmans & Van Tienderen, 2004) to assign pups to their colony or cluster. The program uses the leave‐one‐out (LOO) validation procedure in which an individual to be assigned is removed from its source population and treated as an individual of unknown population origin before calculation of the population allele frequency. The purpose of the LOO procedure is to remove a bias present when allele frequencies are calculated from the same individuals that are subsequently assigned. We used the home likelihood criterion (the likelihood that an individual comes from the population where it was sampled) because it is more appropriate when only part of all possible source populations have been sampled (Meirmans & Van Tienderen, 2004). We used a significance threshold of 0.05 and replaced zero frequencies by 0.005, as suggested by Meirmans and Van Tienderen (2004) and generated 10,000 permutations (i.e., number of datasets). Missing values were replaced by randomly picking alleles from the global allele pool. Assignment power was estimated by calculating the proportion of pups that were correctly assigned to their colony or cluster of origin using (a) the entire dataset of 26,026 SNPs, (b) the subset of 5,000 randomly selected SNPs, and (c) the loci identified as outliers in LOSITAN and Bayescan 2.0.

## 3 RESULTS

### 3.1 Data filtering

Out of a total of 169 genotyped individuals, we excluded two individuals for having more than 90% of loci missing. Out of a total 180,303 biallelic SNPs, we excluded 97,339 SNPs for being missing in at least 80% of individuals, and 56,937 SNPs with <5% minor allele frequency. We further excluded a total of 3,834 SNPs because they were either out of HWE, or in LD. After applying these filters, our dataset consisted of 167 individuals with data for 26,026 SNPs.

### 3.2 Genetic diversity and population structure

Measures of heterozygosity were similar across colonies and regions (Table 2) and *F*_IS_ values were low and only significantly different from zero in one population (WP). Overall, *F*_ST_ values were low (0.003–0.022) and mostly significantly different from zero (*p* < 0.05) between each pair of colonies (27 out of 28; Table 3). NZ‐North‐West (OBI, NP, WP) colonies and NZ‐South (HB, VB, NP) colonies had lower pairwise *F*_ST_ values when compared with each other than with other regions (Table 3). The NZ‐North‐East (CP, OP) colony Ohau Point (OP) had relatively low pairwise *F*_ST_ values when compared with every other colony in this study, especially the NZ‐South colonies. Conversely, Cape Palliser (CP), had relatively high pairwise *F*_ST_ values when compared with every other colony except Ohau Point. The Mantel test revealed a weak, but significant IBD signal (*p* = 0.01, Supporting Information Figure S2). Neither expected heterozygosity estimates nor pairwise *F*_ST_ values were correlated between the microsatellite and SNP datasets (Pearson's *r *=* *0.25, *p* = 0.55; Supporting Information Figure S3a; Mantel's *r*_xy_ = 0.38, *p* = 0.11; Supporting Information Figure S3b).

**Table 2:** Sample sizes, average expected heterozygosity (*H*_e_), average observed heterozygosity (*H*_o_) and *F*_IS_ values with an asterisk depicting values significantly different from zero (*p* < 0.05) after 10,000 permutations for each New Zealand fur seal (*A. forsteri*) cluster and breeding colony

Cluster/Colony	Code	*N*	*H*_o_	*H*_e_	*F*_IS_
NZ‐North‐West		72	0.225	0.259	0.025^*^
Open Bay Islands	OBI	28	0.233	0.267	0.021
Cape Foulwind	CF	23	0.237	0.268	0.009
Wekakura Point	WP	21	0.230	0.268	0.039^*^
NZ‐North‐East		29	0.238	0.263	0.026
Cape Palliser	CP	14	0.247	0.272	0.010
Ohau Point	OP	15	0.250	0.271	0.021
NZ‐South		66	0.230	0.261	0.027^*^
Horseshoe Bay	HB	22	0.227	0.263	0.033
Victory Beach	VB	22	0.246	0.270	0.012
Nugget Point	NP	22	0.246	0.269	0.029

**Table 3:** Pairwise values of Slatkin's linearized *F*_ST_ between New Zealand fur seal (*A. forsteri*) breeding colonies

	OBI	CF	WP	CP	OP	HB	VB	NP
OBI	0							
CF	**0.005**	0						
WP	**0.005**	**0.006**	0					
CP	**0.022**	**0.022**	**0.021**	0				
OP	0.003	**0.010**	**0.009**	**0.020**	0			
HB	**0.016**	**0.017**	**0.017**	**0.023**	**0.010**	0		
VB	**0.013**	**0.014**	**0.014**	**0.020**	**0.011**	**0.006**	0	
NP	**0.015**	**0.016**	**0.014**	**0.019**	**0.011**	**0.004**	**0.005**	** **

Note

Bold values denote significant values following standard Bonferroni correction (*p* < 0.05/*n* tests).

**Figure 2:**
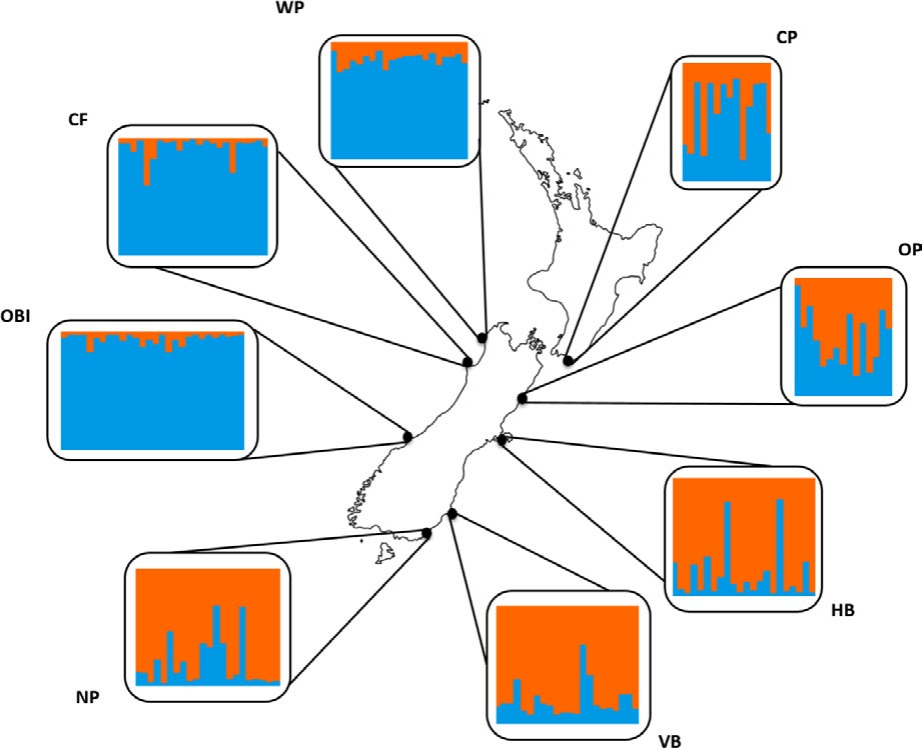
Individual clustering assignment in New Zealand fur seal (*A. forsteri*) (*n* = 167) from eight breeding colonies for the most likely number of clusters (*K* = 2) using 5,000 random SNPs in STRUCTURE [Colour figure can be viewed at http://wileyonlinelibrary.com]

Based on LnP(*K*) and the ad‐hoc statistic ∆*K*, Bayesian clustering analysis for 167 NZFS implemented in STRUCTURE identified two main clusters along a west to east genetic gradient (Figure 2, Supporting Information S4a, S5). NZ‐North‐West (OBI, CF, WK) and NZ‐South (HB, VB, NP) colonies showed low admixture (*q* ≥ 0.83 and 0.79, respectively) while NZ‐North‐East colonies (CP and OP) showed more pronounced admixture (*q* = 0.59 and 0.51). While ADMIXTURE and STRUCTURE results were consistent in describing a west to east genetic gradient, ADMIXTURE supported a single cluster (i.e. lowest cross‐validation error for *K* = 1, Supporting Information Figure S4b, S5).

**Figure 3:**
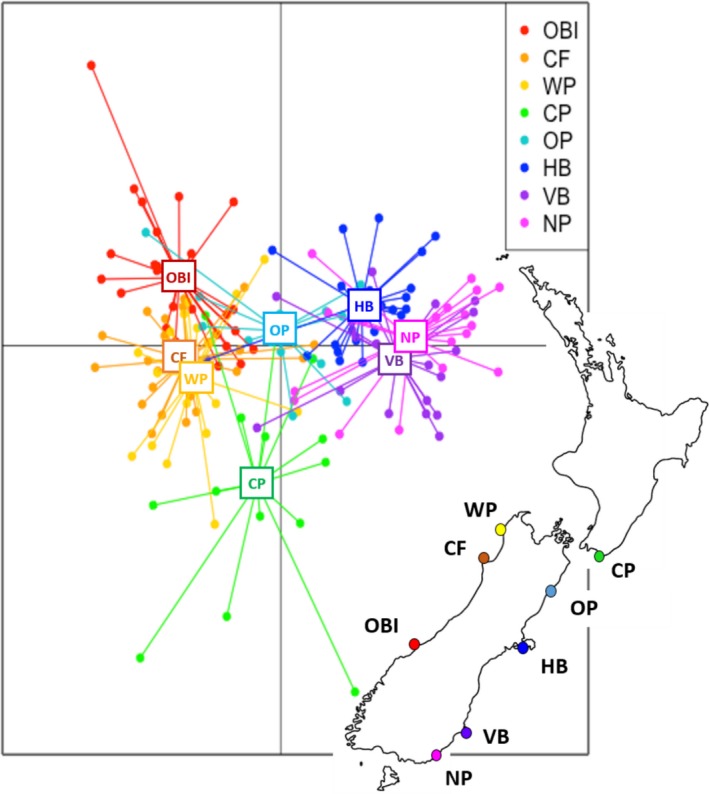
DAPC for 167 New Zealand fur seal (*A. forsteri*) from eight breeding colonies based on 26,026 SNP loci [Colour figure can be viewed at http://wileyonlinelibrary.com]

Discriminant analysis of principal components results were consistent with STRUCTURE and also supported a NZ‐North‐West and a NZ‐South cluster (Figure 3). NZ‐North‐East (CP and OP) colonies shared principal component similarity with both of these clusters, though CP was more divergent than OP from other colonies. AMOVAs based on STRUCTURE and DAPC results for a NZ‐North (OBI, NP, WP, CP, OP) vs. NZ‐South (HB, VB, NP) grouping (Supporting Information Table S1a) and a NZ‐North‐West (OBI, NP, WP) vs. NZ‐North‐East (CP, OP) vs. NZ‐South (HB, VB, NP) grouping (Supporting Information Table S1b) both indicated that 98% of variance was explained by variation among individuals. Only ~0.60% was explained by variation among regions.

### 3.3 Demographic history

Demographic reconstructions using the Approximate Bayesian Computation (ABC) approach strongly supported a scenario of Polynesian‐ and European‐induced declines followed by range‐wide recolonization and population divergence with a posterior probability of 1. Our estimates supported severe bottlenecks with potential 10‐fold and 250‐fold declines in response to Polynesian and European hunting, respectively (Table 1). The observed data summary statistics for the preferred model were included in the confidence interval drawn from pseudo‐observed data. Type I and II errors were of 0.04 and 0.05, respectively.

### 3.4 Outlier loci detection

Using the Fdist2 method implemented in LOSITAN, we did not find any outlier loci under putative selection. The Bayescan method identified 74 colony‐specific and 18 genetic cluster‐specific outlier SNP loci putatively under diversifying selection when analysing the six colonies and East and West Coast clusters, respectively (Supporting Information Table S2). For both datasets, all other loci were considered putatively neutral.

### 3.5 Population assignments

We attempted to assign pups treated as unknowns to colonies and identified clusters using the LOO test. Using either all 26,026 SNPs or the 5,000 randomly selected SNPs, we assigned 167 pups to their colonies of origin with, on average, 44.2% and 42.1% accuracy, respectively, though low assignment probabilities to NZ‐North‐East colonies CP (14%–21%) and OP (0%–7%) drove these averages down (Table 4a). We observed the highest assignment probabilities at the colony level for OBI with 96% and 93% correct assignments for the 26,026 SNPs and 5,000 SNPs, respectively. Using outlier loci increased our colony‐level assignment probabilities by around 10%. Average assignment probabilities to colonies using 74 colony‐specific outlier loci where higher on average (53.3%) when considering all colonies and even higher (69%) when excluding CP and OP, which both showed the lowest assignment probabilities (Table 4a).

**Table 4:** Proportions of New Zealand fur seal (*A. forsteri*) pups correctly assigned to (a) their colonies or (b) genetic clusters inferred from Bayesian clustering analyses. Assignments were performed independently using all 26,026 SNPs, 5,000 randomly selected SNPs, 74 outlier loci identified at the colony level, and 18 outlier loci identified at the genetic cluster level

Colony	Code	*N*	26K SNPs	5K SNPs	74 Outlier SNPs
(a)					
Open Bay Islands	OBI	28	0.96	0.93	0.68
Cape Foulwind	CF	23	0.30	0.26	0.61
Wekakura Point	WP	21	0.43	0.43	0.67
Cape Palliser	CP	14	0.21	0.14	0.00
Ohau Point	OP	15	0.00	0.07	0.07
Horseshoe Bay	HB	22	0.50	0.45	0.73
Victory Beach	VB	22	0.41	0.50	0.68
Nugget Point	NP	22	0.73	0.59	0.82
Average		167	0.442	0.421	0.533
Average (CP,OP excluded)		138	0.555	0.527	0.698

Cluster		*N*	26K SNPs	5K SNPs	18 Outlier SNPs
(b)					
NZ‐North‐West		72	0.97	0.96	0.80
NZ‐North‐East		29	0.17	0.13	0.34
NZ‐South		66	0.94	0.95	0.77
Average		167	0.69	0.68	0.64

Individual assignment probabilities of pups to the three genetic clusters (NZ‐North‐West, NZ‐North‐East and NZ‐South) were on average higher (64%–69%; Table 4b) than for assignments at the colony level. As expected, and in line with assignments probabilities to colonies, assignment probabilities were higher for the NZ‐North‐West (80%–97%) and NZ‐South (77%–95%) clusters than for the highly‐admixed NZ‐North‐East cluster (13%–34%; Table 4b) across all SNP datasets. However, the assignment probability to the admixed colonies was highest (34%) when using the 18 outlier loci (Table 4b).

## 4 DISCUSSION

### 4.1 Subtle population structure and near panmixia

Our panel of 26,026 SNPs illustrates the potential of RRS methods to identify previously undetected sources of variation in non‐model species. Overall, our data strongly support a pattern of near panmixia in NZFS, but pairwise *F*_ST_ values among range‐wide colonies supported a weak but significant west to east genetic gradient. Bayesian clustering and DAPC results support the existence of two main genetic clusters (NZ‐North‐West and NZ‐South), and further suggest that NZ‐North‐East colonies (CP and OP) are sites of genetic admixture between these two main clusters. This observed structure was further supported by constructing a PCA from a matrix of estimated genomic relationships (Patterson, Price, & Reich, 2006), which in turn were calculated following the bioinformatics and statistical methods in Dodds et al. (2015). This approach allows the PCA to be formed when there are missing genotype calls and without the use of stringent filtering as the method directly accounts for the read depth and missing observations and thus gives further support to our results.

The weak IBD pattern identified in the Mantel test is consistent with previous observations of highly migratory pinniped species undergoing post‐bottleneck recolonizations (e.g., *Arctocephalus gazella*, Hoffman et al., 2011), as well as of other marine mammals with comparable vagility (e.g., dusky dolphin, Harlin, Markowitz, Baker, Würsig, & Honeycutt, 2003; common dolphin, Stockin, Amaral, Latimer, Lambert, & Natoli, 2014). However, it is crucial to remain cautious when interpreting such a weak IBD pattern, especially when the underlying *F*_ST_ values are generated with tens of thousands of SNPs. As the significance of *F*_ST_ values is likely a result of the size and power of the dataset (Helyar et al., 2011; Morin, Martien, & Taylor, 2009), we stress that various lines of evidence in the form of additional analyses should be considered when interpreting such data, especially when the pattern observed is not clear‐cut and where there is ample room for interpretation. Here, we achieve this goal using STRUCTURE, ADMIXTURE, DAPC, AMOVA and genetic assignments and conclude that NZFS breeding colonies are not markedly genetically different from each other.

### 4.2 Performance of SNPs loci

*H*_e_ and *H*_o_ values did not differ substantially among colonies (0.225–0.250), which is consistent with previous results based on microsatellite data (Dussex et al., 2016; Robertson & Gemmell, 2005) with *H*_e_ and *H*_o_ between (0.67–0.79). Higher estimates of *H*_e_ derived from microsatellite markers may be a consequence of ascertainment bias caused by selecting the most polymorphic markers (Haasl & Payseur, 2011; Queirós et al., 2015). However, there was no significant correlation between *H*_e_ or *F*_ST_ estimates derived from microsatellite and the SNPs generated here. The lack of correlation between *H*_e_ estimates could be due to low sample size (e.g., Fischer et al., 2017) while for pairwise *F*_ST_, it could also be due to an ascertainment bias in the selection of microsatellite loci (Haasl & Payseur, 2011; Ryynänen, Tonteri, Vasemägi, & Primmer, 2007).

In spite of this very subtle population structure, assignment testing results indicate that large SNP marker sets allow NZFS pups to be assigned to their geographic regions of origin with high confidence, but less so to specific colonies. The proportion of pups correctly assigned to genetic clusters (64%–69%) and to breeding colonies (42%–53%) were on average higher for all SNP datasets used here than for microsatellite markers (59.3% and 22.7% correctly assigned to clusters and colonies, respectively) (Dussex et al., 2016; Robertson & Gemmell, 2005). Furthermore, a random subset of 5,000 SNPs was roughly as effective as the entire 26,026 SNP dataset in achieving these assignments, while using outlier loci in some instances increased the proportion of pups correctly assigned to their colony or genetic cluster of origin. As expected, the proportion of correct assignments was lowest for the NZ‐North‐East cluster and its colonies (CP and OP), which is in line with the identification of a zone admixture between the NZ‐North‐West and NZ‐South clusters.

Our higher assignment probabilities can likely be attributed to the higher statistical power of our dataset (Helyar et al., 2011; Morin, Luikart, & Wayne, 2004; Morin et al., 2009), although we were still unable to achieve confident assignment to specific colonies. One exception is that of OBI, which also had one if the highest proportion of correct assignments with microsatellite data (68.8%; Dussex et al., 2016). This result is in line with the suggestion that OBI may represent a refugium from sealing (Dussex et al., 2016; Salis et al., 2016; Smith, 2005) and thus a colony with a higher proportion of private alleles, thus increasing the proportion of pups correctly assigned. While colony‐level assignment for species may be unfeasible with the tools presently available, our results suggest that outlier loci assignment techniques could enable confident assignment in the future (e.g., Nielsen et al., 2012). However, identifying highly differentiated outlier loci will be crucial for accurate assignments as genetic structure is predicted to become rapidly eroded due to the high vagility of the species.

As a whole, our measures of intra and interspecific population structure and assignments are consistent with what might be expected from the severe historic bottleneck and rapid range‐wide recolonization NZFS experienced, a model which has been confirmed through Bayesian reconstruction here and in previous studies (Dussex et al., 2016; Emami‐Khoyi et al., 2016).

### 4.3 Demographic recovery and transient population structure

The subtle population structuring seen here for NZFS is most likely due to the high vagility of the species and its recolonization history (Dussex et al., 2016; Emami‐Khoyi et al., 2017). Demographic reconstructions strongly supported a scenario of Polynesian‐ and European‐induced declines followed by range‐wide recolonization and population divergence with a posterior probability of 1, which is consistent with archaeological records (Smith, 1989) and the recent exploitation history of the species (Lalas & Bradshaw, 2001). We estimated that a very small population survived European sealing with an *N*_e_ of ~110 (90%HPD 102–161), which is consistent with Emami‐Khoyi et al. (2017) who estimated a bottleneck *N*_e_ of 116. However, we estimated much larger pre‐European and pre‐Polynesian *N*_e_ of ~570,000 and ~4,900,000, which is one to two orders of magnitude higher than previous estimates of historical *N*_e_ (Dussex et al., 2016; Emami‐Khoyi et al., 2017). Our contemporary *N*_e_ estimates seem more realistic with an *N*_e_ of 600–1,000 predicted following demographic recovery after cessation of sealing. The estimate for the post‐European surviving population seems extremely low, yet not unlikely based on the history of near extermination of this and other fur seal species in the 1830s (Lalas & Bradshaw, 2001). For instance, only 5 NZFS were reported in the Bounty Islands in the 1830s (Taylor 1982). On the mainland, it has been estimated, colonies could have been reduced to <2% of their original size over 25–35 years (Lalas & Bradshaw, 2001), making hunting unprofitable and the possibility of few remaining breeders very likely. Moreover, preliminary runs comparing a model including a genetic bottleneck associated with Europeans and an alternative model that did not consider a genetic bottleneck, supported the former model, suggesting that a genetic bottleneck did actually occur. One explanation for the very low *N*_e_ in this surviving population compared to the larger *N*_e_ in modern populations may be due to unsampled populations that might have contributed genetic diversity to the modern populations and possibly range expansions of other species and, as yet, undetected hybridization (Lancaster et al., 2006). In fact, the main breeding locations in New Zealand in the 1970s comprised the Fiordland coastline at the south‐western corner of South Island and the Chatham Islands, as well as islands in the Foveaux Strait, between Stewart Island and the South Island (Crawley, 1990; Wilson, 1981). It is thus possible that gene flow from unsampled colonies in Foveaux Strait, or subantarctic islands, may have rapidly inflated the *N*_e_ of modern populations.

While theory predicts that demographic decline can result in population structuring via the effects of genetic drift (Charlesworth, 2009; Frankham, 2005), NZFS as well as other otariids do not show marked population structuring (Dussex et al., 2016; Hoffman et al., 2011). This could be due to the fact that the demographic bottleneck in NZFS was too short in duration to induce a significant loss of genetic diversity and strong population differentiation via genetic drift and to a high dispersal rate contributing to range wide gene flow and homogenization of allelic distribution. NZFS most likely survived in refugia post‐exploitation, and quickly recolonized after sealing became unprofitable (Dussex et al., 2016; Emami‐Khoyi et al., 2017). Therefore, the lack of marked population structuring is most likely due to the high vagility of the species and its recolonization history. It is even possible that the subtle structure detected here is transient and that the whole species may represent a panmictic unit within a few generations because population size has increased dramatically in the years since official the cessation of seal hunting (Baird, 2011). Consequently, genetic assignments may become more difficult in the future and may require more traditional approaches, such as tagging and mark‐recapture to assign bycatch to colonies (e.g., Bradshaw, Davis, Lalas, & Harcourt, 2000).

## 5 CONCLUSION

To date, few studies have compared the performance of microsatellite and SNPs for highly vagile species. Overall, while the genetic structure described here was not substantially different from that examined with microsatellite loci, a larger number of markers allowed for better assignments of pups to their genetic cluster and colony of origin. This was especially true for colonies that were outside of the zone of admixture.

Our results, however, suggest that because of the ascertainment bias associated with the selection of microsatellite loci, over‐estimation of population structuring is possible when using such markers. SNP loci are thus not only less biased but also offer more statistical power. Nevertheless, due to the species’ recolonization history and high vagility, describing finer‐scale structuring remains challenging.

Recent advances in whole genome sequencing present new avenues for research in NZFS. For instance, hunting may have impacted fur seal functional diversity, either via the effects of drift or via artificial selection pressures imposed by hunting. While the effective population size may not have been significantly affected, modern functional genetic diversity may not be representative of the prehuman functional diversity as the shift of genetic composition in southern New Zealand since human arrival suggests (Salis et al., 2016). Comparison of pre‐ and post‐hunting functional genetic diversity would thus provide insights in the potential effects of hunting as artificial selection pressure.

## CONFLICT OF INTEREST

None declared.

## AUTHORS’ CONTRIBUTIONS

N. Dussex designed research, performed population structure analyses, and wrote main body of text. H.R. Taylor advised on population genetic theory and lab procedures, analysed sequencing output, reviewed, and contributed to writing the paper. W. R. Stovall designed research, performed lab work and bioinformatics processing of data, performed population structure analyses, and wrote main body of text. K. Rutherford performed bioinformatics processing of data, analysed sequencing output, and oversaw data management and archiving. K. G. Dodds advised on visualization of results, and assisted with post‐sequencing data management. S. M. Clarke assisted with formulation of project, performed GBS and Illumina sequencing, and reviewed and contributed to writing the paper. N.J. Gemmell designed research, performed and oversaw field sampling, and supervised project. All authors contributed substantially to the writing and refinement of the final text.

## DATA ARCHIVING STATEMENT

Raw sequence data, processed data and scripts are available from the Dryad Digital Repository: https://doi.org/10.5061/dryad.63nm407.
