# Supplementary material for: Reduced representation sequencing detects only subtle regional structure in a heavily exploited and rapidly recolonizing marine mammal species
Source: Ecol Evol. 2018 Aug 5;8(17):8736–49. doi: 10.1002/ece3.4411 (PMC6157699; doi:10.1002/ece3.4411)
Supplement: Supplementary file 1 [file ECE3-8-8736-s001.docx]

# Supplementary Tables and Figures

### **Table S1** a) AMOVA results for a 2-clusters grouping: NZ-North (OBI, NP, WP, CP, OP) and NZ-South (HB, VB, NP) and b) for a 3-clusters grouping: NZ-North-West (OBI, NP, WP), NZ-North-East (CP, OP) and NZ-South (HB, VB, NP).

| 1. **2 clusters** | | | | |
| --- | --- | --- | --- | --- |
| **Source of Variation** | **d.f.** | **Sum of Squares** | **Variance Components** | **Percentage of Variation** |
| **Among clusters** | 1 | 556.521 | 1.372 | 0.60 |
| **Among populations within clusters** | 6 | 1951.938 | 2.193 | 0.97 |
| **Within populations** | 326 | 80981.906 | 223.706 | 98.43 |
| **Total** | 369 | 83490.365 | 227.273 |  |
|  |  |  |  |  |
| 1. **3 clusters** | | | | |
| **Source of Variation** | **d.f.** | **Sum of Squares** | **Variance Components** | **Percentage of Variation** |
| **Among clusters** | 2 | 691.419 | 1.194 | 0.65 |
| **Among populations within clusters** | 5 | 1119.073 | 0.985 | 0.53 |
| **Within populations** | 326 | 59333.690 | 182.005 | 98.82 |
| **Total** | 333 | 61144.183 | 184.377 |  |

### **Table S2** (a) Outlier loci identified using Bayescan 2.0 (Foll & Gaggiotti, 2008) at the colony level (OBI, NP, WP, CP, OP, HB, VB, NP; 74 loci) and (b) at the cluster level (NZ-North-West, NZ-North-East and NZ-South; 18 loci). The alpha value indicates the direction of selection with positive values suggesting diversifying selection and negative values suggesting balancing or purifying selection. The q-value corresponds to the false discovery rate (FDR) of 0.05 as a threshold for outlier loci detection.

| **(a)** | | |  |  |
| --- | --- | --- | --- | --- |
| **Locus ID** | ***F*_ST_** | **alpha** | | **q-value** |
| 2874_12 | 0.078299 | 1.7768 | | 0.0047282 |
| 3430_12 | 0.055809 | 1.3613 | | 0.045258 |
| 7470_63 | 0.056755 | 1.4145 | | 0.024361 |
| 13350_27 | 0.08263 | 1.8147 | | 0.0063397 |
| 15901_56 | 0.068936 | 1.6607 | | 0.0041808 |
| 17418_67 | 0.064671 | 1.4947 | | 0.034564 |
| 18050_50 | 0.056393 | 1.3807 | | 0.043 |
| 21858_87 | 0.079195 | 1.7747 | | 0.0035785 |
| 21858_89 | 0.070976 | 1.6056 | | 0.019422 |
| 22405_21 | 0.073797 | 1.6898 | | 0.010826 |
| 26529_15 | 0.067457 | 1.6139 | | 0.014362 |
| 27269_32 | 0.067021 | 1.538 | | 0.03673 |
| 27754_59 | 0.073912 | 1.7401 | | 0.0024433 |
| 33074_14 | 0.066349 | 1.6006 | | 0.0089889 |
| 33647_43 | 0.057324 | 1.4047 | | 0.039814 |
| 36046_14 | 0.060763 | 1.4624 | | 0.038805 |
| 39161_12 | 0.065745 | 1.5928 | | 0.012208 |
| 42913_72 | 0.054584 | 1.3485 | | 0.047476 |
| 47794_5 | 0.058426 | 1.4474 | | 0.027205 |
| 50540_71 | 0.061673 | 1.4688 | | 0.033464 |
| 55380_86 | 0.085491 | 1.8545 | | 0.0029881 |
| 55380_91 | 0.084636 | 1.8446 | | 0.003885 |
| 55530_32 | 0.055367 | 1.37 | | 0.041847 |
| 57073_65 | 0.074325 | 1.7015 | | 0.0068532 |
| 57240_9 | 0.062106 | 1.4854 | | 0.031297 |
| 57376_5 | 0.070515 | 1.6103 | | 0.026255 |
| 58244_13 | 0.070784 | 1.6536 | | 0.012916 |
| 62496_38 | 0.065053 | 1.5454 | | 0.026255 |
| 64497_91 | 0.065461 | 1.5088 | | 0.044142 |
| 65224_79 | 0.091604 | 1.933 | | 0.0006668 |
| 67526_60 | 0.065537 | 1.5705 | | 0.015213 |
| 68121_24 | 0.066233 | 1.6104 | | 0.0078912 |
| 69099_89 | 0.088086 | 1.9307 | | 0.00020004 |
| 69327_37 | 0.07635 | 1.7746 | | 0.0015803 |
| 70526_6 | 0.073429 | 1.7409 | | 0.0011252 |
| 72634_56 | 0.059257 | 1.4559 | | 0.023364 |
| 74220_69 | 0.077333 | 1.7581 | | 0.0054344 |
| 74825_89 | 0.064422 | 1.5114 | | 0.032351 |
| 82581_90 | 0.07436 | 1.6805 | | 0.017631 |
| 83860_23 | 0.075236 | 1.7538 | | 0.0015803 |
| 87827_23 | 0.070032 | 1.6372 | | 0.013645 |
| 87952_23 | 0.084448 | 1.8435 | | 0.0073658 |
| 88654_61 | 0.071784 | 1.5961 | | 0.040835 |
| 88779_42 | 0.059951 | 1.4534 | | 0.035656 |
| 88873_26 | 0.082686 | 1.8078 | | 0.0084417 |
| 89590_44 | 0.059259 | 1.4738 | | 0.018508 |
| 89637_90 | 0.088323 | 1.8911 | | 0.0020171 |
| 90703_83 | 0.072085 | 1.7063 | | 0.0022158 |
| 91508_11 | 0.074349 | 1.7382 | | 0.0027072 |
| 92071_14 | 0.059459 | 1.4664 | | 0.020387 |
| 94188_86 | 0.068883 | 1.6584 | | 0.004458 |
| 101398_63 | 0.059955 | 1.4791 | | 0.022359 |
| 103222_31 | 0.085509 | 1.8937 | | 0.0004801 |
| 106370_18 | 0.062548 | 1.4995 | | 0.02822 |
| 107623_11 | 0.10013 | 2.0997 | | 0 |
| 108545_69 | 0.069146 | 1.6318 | | 0.011517 |
| 108602_35 | 0.086205 | 1.8504 | | 0.010196 |
| 110249_26 | 0.092309 | 2.0143 | | 0 |
| 111178_45 | 0.078775 | 1.8094 | | 0.00091447 |
| 114051_31 | 0.059486 | 1.4112 | | 0.04981 |
| 120516_48 | 0.063031 | 1.5422 | | 0.016813 |
| 122355_82 | 0.056375 | 1.4029 | | 0.029235 |
| 123630_20 | 0.078392 | 1.7848 | | 0.0050097 |
| 123737_75 | 0.059027 | 1.3962 | | 0.046367 |
| 123881_28 | 0.080016 | 1.8253 | | 0.0020171 |
| 126150_87 | 0.070629 | 1.627 | | 0.016813 |
| 128497_26 | 0.073336 | 1.6978 | | 0.0095707 |
| 129999_91 | 0.065279 | 1.5175 | | 0.037782 |
| 132182_53 | 0.067262 | 1.6263 | | 0.0058652 |
| 137196_14 | 0.063997 | 1.4757 | | 0.04864 |
| 139234_7 | 0.062795 | 1.5174 | | 0.021353 |
| 139317_30 | 0.070527 | 1.683 | | 0.0032948 |
| 147894_25 | 0.064148 | 1.5136 | | 0.030224 |
| 154258_51 | 0.089767 | 1.9407 | | 0.00035007 |
| **(b)** |  |  | |  |
| **Locus ID** | ***F*_ST_** | **alpha** | | **q-value** |
| 13350_27 | 0.062008 | 1.6176 | | 0.032116 |
| 33074_14 | 0.056317 | 1.5644 | | 0.013936 |
| 33647_43 | 0.053344 | 1.4953 | | 0.03399 |
| 42190_6 | 0.059827 | 1.5692 | | 0.042046 |
| 68121_24 | 0.049071 | 1.3849 | | 0.048221 |
| 69099_89 | 0.057953 | 1.59 | | 0.016753 |
| 72634_56 | 0.053908 | 1.5146 | | 0.025091 |
| 82581_90 | 0.063694 | 1.6553 | | 0.02683 |
| 83860_23 | 0.062063 | 1.6737 | | 0.0085017 |
| 88873_26 | 0.065988 | 1.6958 | | 0.020084 |
| 94188_86 | 0.05455 | 1.5204 | | 0.022971 |
| 106370_18 | 0.054608 | 1.5011 | | 0.036023 |
| 107248_34 | 0.06162 | 1.6023 | | 0.038093 |
| 107623_11 | 0.061631 | 1.6776 | | 0.0084017 |
| 110249_26 | 0.054931 | 1.523 | | 0.030166 |
| 115714_54 | 0.050137 | 1.3993 | | 0.045233 |
| 123586_59 | 0.063218 | 1.6436 | | 0.028495 |
| 123881_28 | 0.053535 | 1.4835 | | 0.040008 |

**Figure S1** Demographic scenarios implemented in DIYABC for New Zealand fur seal *A. forsteri* 1000 SNP loci (n=167) with Scenario 1 including a Polynesian- and European-induced decline followed by recolonisation and population divergence and Scenario 2 assuming a constant population size through time followed by population divergence.

### **
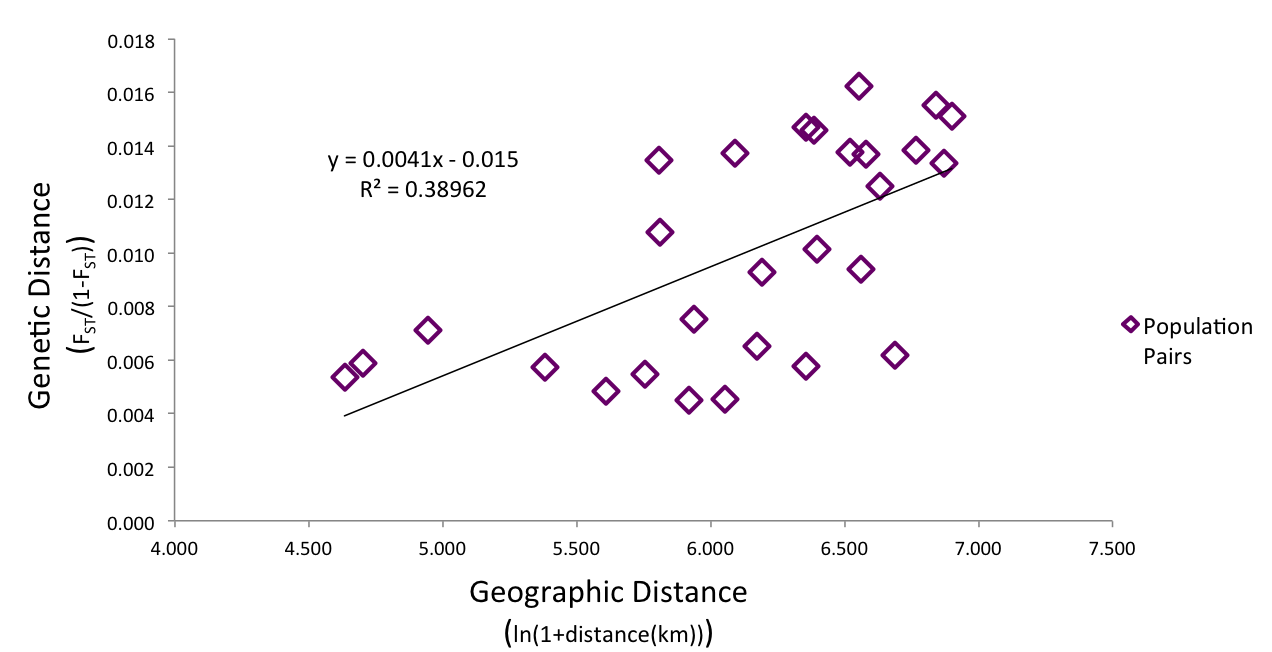
**

### **Figure S2** Mantel test of correlation between geographic and genetic distance for New Zealand fur seal *A. forsteri* based on 26,026 SNP loci. The isolation by distance pattern detected is weak (y=0.0041x – 0.015), but significant (p=0.01).

(a)

(b)

**Figure S3.** Association between 26,026 bi-allelic SNP loci and 11 microsatellites loci (Dussex et al. 2016) for (a) heterozygosity (H_e_) (Pearson’s *r* = 0.25, p = 0.55) and (b) Pairwise *F*_ST_ (Mantel’s *r*_xy_ = 0.38, P = 0.11) estimates from eight New Zealand fur seal *A. forsteri* colonies.

**Figure S4**. (a) Individual clustering assignment in New Zealand fur seal *Arctocephalus forsteri* (n = 167) from 8 breeding colonies for K = 2-8 using 5,000 random SNPs in STRUCTURE and (b) using all 26,026 SNP loci ADMIXTURE.

(a)

(b)

### **Figure S5.** (a) Results of STRUCTURE analyses with the Mean estimated log-normal (Ln) probability of the data in relation to the simulated number of clusters K where vertical bars indicate the standard deviation among ten replicates and the Delta K (Evanno et al. 2005) in relation number of clusters K. (b) Cross-validation error when testing for the most likely number of clusters K in ADMIXTURE (Alexander et al. 2009).
